# Managing surgical demand when needs outstrip resource: qualitative investigation of colorectal cancer surgery provision in the first wave of the COVID-19 pandemic

**DOI:** 10.1093/bjs/znac371

**Published:** 2022-11-07

**Authors:** Carmel Conefrey, Cynthia Ochieng, Christin Hoffmann, Daisy Elliott, Kerry Avery, Joanne Bennett, Natalie Blencowe, Sarah Duff, James Kinross, Angus McNair, David Messenger, Anne Pullyblank, Baljit Singh, Anni King, Sarah E Squire, Jane Blazeby, Barry Main, Leila Rooshenas

**Affiliations:** Population Health Sciences, Bristol Medical School, University of Bristol, Bristol, UK; NIHR Bristol Biomedical Research Centre at University Hospitals Bristol and Weston NHS Foundation Trust and the University of Bristol, Bristol, UK; Bristol Centre for Surgical Research, Population Health Sciences, Bristol Medical School, University of Bristol, Bristol, UK; NIHR Bristol Biomedical Research Centre at University Hospitals Bristol and Weston NHS Foundation Trust and the University of Bristol, Bristol, UK; Bristol Centre for Surgical Research, Population Health Sciences, Bristol Medical School, University of Bristol, Bristol, UK; NIHR Bristol Biomedical Research Centre at University Hospitals Bristol and Weston NHS Foundation Trust and the University of Bristol, Bristol, UK; Bristol Centre for Surgical Research, Population Health Sciences, Bristol Medical School, University of Bristol, Bristol, UK; NIHR Bristol Biomedical Research Centre at University Hospitals Bristol and Weston NHS Foundation Trust and the University of Bristol, Bristol, UK; Bristol Centre for Surgical Research, Population Health Sciences, Bristol Medical School, University of Bristol, Bristol, UK; Department of General Surgery, Gloucestershire Royal Hospitals NHS, Gloucester, UK; NIHR Bristol Biomedical Research Centre at University Hospitals Bristol and Weston NHS Foundation Trust and the University of Bristol, Bristol, UK; Bristol Centre for Surgical Research, Population Health Sciences, Bristol Medical School, University of Bristol, Bristol, UK; Department of Surgery, Manchester University NHS Foundation Trust, Manchester, UK; Imperial College Healthcare NHS Trust, London, UK; NIHR Bristol Biomedical Research Centre at University Hospitals Bristol and Weston NHS Foundation Trust and the University of Bristol, Bristol, UK; Department of Gastrointestinal Surgery, North Bristol NHS Trust, Bristol, UK; Department of Coloproctology, University Hospitals Bristol and Weston NHS Foundation Trust, UK; Department of Surgery, North Bristol NHS Trust, Bristol, UK; Department of Colorectal Surgery, University Hospitals of Leicester NHS Trust, Leicester, UK; NIHR Bristol Biomedical Research Centre at University Hospitals Bristol and Weston NHS Foundation Trust and the University of Bristol, Bristol, UK; Bristol Centre for Surgical Research, Population Health Sciences, Bristol Medical School, University of Bristol, Bristol, UK; Association of Coloproctology of Great Britain and Ireland Patient Liaison Group, Oxford, UK; NHS Specialised Colorectal Clinical Reference Group, UK; NIHR Bristol Biomedical Research Centre at University Hospitals Bristol and Weston NHS Foundation Trust and the University of Bristol, Bristol, UK; Bristol Centre for Surgical Research, Population Health Sciences, Bristol Medical School, University of Bristol, Bristol, UK; Bristol Dental School, University of Bristol, Bristol, UK; Population Health Sciences, Bristol Medical School, University of Bristol, Bristol, UK; Bristol Centre for Surgical Research, Population Health Sciences, Bristol Medical School, University of Bristol, Bristol, UK

## Abstract

**Background:**

At the onset of the COVID-19 pandemic, elective surgical provision was severely affected by the need for hospital reorganization to care for critically ill patients. In response, National Health Service (NHS) England issued national guidance proposing acceptable time intervals for postponing different types of surgical procedure. This study reports healthcare professionals’ private accounts of the strategies adopted to manage the imbalance of demand and resource, using colorectal cancer surgery as a case study.

**Methods:**

Twenty-seven semistructured interviews were conducted with healthcare professionals between June and November 2020. A key informant sampling approach was used, followed by snowballing to achieve maximum regional variation across the UK. Data were analysed thematically using the constant comparison approach.

**Results:**

In the context of considerable resource constraint, surgical teams overcame challenges to continue elective cancer provision. They achieved this by pursuing a combination of strategies: relocating surgical services; prioritizing patients within and across surgical specialties; adapting patient treatment plans; and introducing changes to surgical team working practices. Despite national guidance, prioritization decisions were framed as complex, and the most challenging of the strategies to implement, both practically and emotionally.

**Conclusion:**

There is a need to better support surgeons tasked with prioritizing patients when capacity exceeds demand.

## Teaser

Using colorectal cancer surgery as a case study, this qualitative study represents the first in-depth investigation of how surgical teams managed to uphold surgical services in the first wave of the COVID-19 pandemic, despite contending with immense resource constraint. The findings revealed a combination of strategies pursued, including: relocating surgical services; prioritizing patients within and across surgical specialties; adapting patient treatment plans; and introducing changes to surgical team working practices. It was concluded that surgeons need to be better supported to manage the task of prioritizing patients, and raises wider questions about who should be involved in this key activity.

## Introduction

The start of the COVID-19 pandemic in 2020 triggered reorganization of hospital departments around the world. Resources were directed towards treating patients with COVID-19, leading to cancellation of elective surgery across all specialties^1^. In England, national bodies (National Health Service (NHS) England) recommended that hospitals postpone non-urgent elective surgery for at least 3 months, although cancer treatment was to ‘continue unaffected’^2^. In an attempt to maintain some capacity for cancer treatment, NHS leaders secured the use of independent-sector hospitals^3^. Guidance from NHS England outlined several prioritization categories, to help clinicians plan provision of cancer operations^4^. Categories ranged from P1a (surgery within 24 h) to P3 (surgery can be delayed for 10–12 weeks). More detailed recommendations followed, specifying the types of cancer procedure that fell into each category^5^.

Recommendations and guidance were intended to help navigate difficult decisions about how to use limited NHS resources, but the implications for practice and patient care were unknown. The CONSIDER-19 study sought to investigate prioritization decisions in practice, against this backdrop of national guidance. Colorectal cancer surgery was selected as a case study to enable in-depth exploration of these issues, given NHS England’s recommendation that most colorectal cancer resections could be delayed by 10–12 weeks or longer. The Association of Coloproctology of Great Britain and Ireland guidance^6^ acknowledged that complex exenteration surgery for certain cancers might not be performed at all. Early guidance also suggested risk-minimizing adaptations (for example avoidance of laparoscopy) and anticipated higher rates of stoma formation^6,7^.

The impact of COVID-19 on the diagnosis and treatment of colorectal cancer has started to be quantified^8,9^, as has adherence to NHS and specialist association guidance^10^. Little, however, is known about the processes of decision-making on the ground, or front-line clinical practitioners’ experiences of implementing national or locally sourced strategies to continue surgical provision under intense resource constraint. This article addresses these evidence gaps by reporting healthcare professionals’ private accounts of the strategies adopted to manage the imbalance between resource and demand provoked by COVID-19, using colorectal cancer surgery as a case study.

## Methods

This was a qualitative study, consisting of in-depth semistructured interviews with healthcare professionals. The University of Bristol Faculty of Health and Sciences Research Ethics Committee approved the study (FREC104342).

### Setting and participants

Eligible participants included clinical professionals responsible for overseeing/delivering surgical care for patients diagnosed with colorectal cancer. This included, but was not limited to, surgeons, clinical nurse specialists, and stoma nurses. A key informant sampling approach was used initially, whereby potential participants were identified by the CONSIDER-19 Study Group, with the intention of achieving maximum geographical variation. The sample was then amplified through snowballing, whereby participants recommended others who met the eligibility criteria.

### Data collection

Four trained qualitative researchers conducted semistructured interviews via telephone/web platforms between June and November 2020. Interviews lasted 40–80 min, were audio-recorded, and fully transcribed. Sampling continued until saturation, where no new relevant insights arose from interviews. A topic guide was used to ensure consistency in questions covered. The first version of the guide was reviewed and adapted following analysis of the first six interviews.

### Analysis

Two researchers analysed the data thematically, using the constant comparison method adopted from grounded theory methodology^11^. This involved applying codes to capture the content and meaning of transcripts and arranging codes iteratively into thematic hierarchies. Four transcripts were coded by two authors independently and discussed to ensure reliability in the early stages of analysis, followed by regular discussion of the analysis as data collection continued. This was facilitated by an evolving descriptive account, which summarized emerging themes, supported with extracts from transcripts to ensure that the findings were grounded in data. The developing themes were also discussed in a stakeholder meeting (December 2020), providing a sense check on emerging findings.

## Results

Interviews took place with 27 informants (22 surgeons, 3 colorectal nurse specialists, 1 stoma nurse, and 1 gastroenterologist), representing 16 hospitals across 8 UK regions and 1 national organization (*Table S1*).

The findings are structured in two sections: part 1 provides contextual information as a foundation for understanding part 2, which presents the strategies surgical teams reported using to continue elective surgical provision during the early surges of COVID-19 infections. Findings are supported with evidence in *Figs S1–S5*, with explicit mention of negative cases (that is exceptions) where relevant. Additional evidence is provided in *Appendix S1*.

### Part 1 (context): capacity for surgery and engagement with national guidance

Between March 2020 and the time of the interview, interviewees from all sites reported a reduction in surgical numbers, primarily owing to fewer patients coming through diagnostic pathways (*Appendix S1*, extract (E) 1). Interviewees attributed these reductions to several factors, including: reductions in general practitioner referrals (*Appendix S1*, E2); interruption to the national bowel screening programme (*Appendix S1*, E3); reduced access to colonoscopies and CT (*Appendix S1*, E4), and perceived patient hesitancy around attending hospital (*Appendix S1*, E5).

Despite perceptions of fewer patients being diagnosed, many informants reported that the demand for surgical services exceeded resources in the early months of the pandemic. Interviewees referred to theatre and recovery space being transformed into ICU provision for patients with COVID-19 (*Appendix S1*, E6), which was compounded by disruption to staffing. Several informants referred to theatre, nursing, and junior trainees being redeployed (*Appendix S1*, E7 and E8), and rotas being revised to cope with staff shielding (*Appendix S1*, E9). This combination of theatre and staffing constraints reportedly led to a pause in elective colorectal surgery. At one extreme, one informant referred to a 3-month cessation of surgery; at the other end of the spectrum, some reported that surgery continued without cessation, with only a minimal reduction in operating activity. Others reported a pause in surgery, ranging from 4 to 10 weeks.

Most interviewees framed their management of surgical care with reference to NHS England, Royal Colleges, and specialty association guidance, although perspectives on the role of this guidance varied. Some reported adhering to guidance closely (*Fig. S1*, E1), whereas others framed guidance more liberally, appreciating the agency and sensitivity to local context it afforded (*Fig. S1*, E2). Not all interviewees welcomed the ability to localize, with one informant expressing concern about varying implementation between neighbouring Trusts (*Fig. S1*, E3). Although acknowledging the difficult circumstances for developing guidance, there were concerns that it was overly reliant on individual experience rather than robust evidence (*Fig. S1*, E4), and that a lack of coordination between the Royal Medical Colleges and associations had resulted in voluminous, misaligned guidance (*Fig. S1*, E5). As such, several interviewees reported having to pick and choose from the various guidelines in line with their own experience and interpretation of the evidence (*Fig. S1*, E6).

#### Summary of contextual findings

Informants experienced a spectrum of disruption to services in the early phase of the pandemic, and expressed mixed views about the value of national guidance. Building on this, surgeons and nurses discussed a range of strategies they had adopted to manage the impact of COVID-19 on their practice. Some of these were related to national guidance, with adaptations to local context, whereas others were framed as locally sourced solutions. These strategies are discussed hereafter.

### Part 2: strategies for continuing surgical management of patients

Interviewees’ accounts revealed four strategies adopted to enable continued provision of colorectal cancer surgery in the face of COVID-19-related resource constraint. These were: relocation of surgical services; prioritization within and across specialties; adapting patient treatment plans; and changing surgical team working practices. These strategies, including their associated challenges and solutions, are discussed below.

#### Relocation of surgical services

Informants from one-quarter of sites (4) drew on the independent sector and a neighbouring NHS hospital to relocate some/all surgical practice given limited capacity in their own hospitals. Relocation was not challenge-free. Interviewees reported unfamiliarity with site-specific policies and practices, such as tensions arising from differing personal protective equipment policies (*Fig. S2*, E1).

Different thresholds for operating were also reported, as informants reflected on the variation in the types of patient admitted for surgery in their hospital compared with independent-sector hospitals. One informant discussed how the independent hospital they referred to had more restrictive criteria, as reflected in their experience of patients being turned down (*Fig. S2*, E2).

In a similar vein, an informant from a different site reported how they selectively referred patients whom they considered to be at lower risk of complications, given concern about independent-sector hospitals’ limited ICU provision and skilled nursing (*Fig. S2*, E3). With time, informants became more comfortable referring patients without selection, as independent providers adapted by expanding ICU provision, upskilling their existing staff, and/or were joined by colorectal nursing staff from NHS sites (*Fig. S2*, E4).

All interviewees with experience of relocating surgery to independent-sector hospitals reported this had been useful for expanding capacity. For the majority, however, relocation was not an option, largely owing to lack of ICU facilities and insufficient staff to support both on-site and independent-sector provision. Many informants considered such provision more suited to smaller day procedures, often citing breast and skin cancer surgery. A single surgeon mentioned the option of transferring patients and their surgical care to an NHS regional cancer surgery hub. They voiced reluctance, considering it not in their patients’ interests, preferring local care, even during a brief interval where surgery was paused (*Fig. S2*, E5).

Despite the NHS securing independent-sector capacity, most surgical teams reported pursuing other strategies to optimize on-site facilities and staffing resources.

#### Prioritization within and across specialties

Informants widely reported using NHS guidance on prioritization. There was, however, a need to make judgements about which category was most appropriate for each patient. This was framed by many as a new kind of decision-making in surgical practice, which some framed as uncomfortable (*Fig. S3*, E1).

Typically, interviewees presented decision-making about who was listed for surgery as a multistage process, comprising: assigning patients to a priority group within their own specialty; presenting their prioritized patients at cross-specialty meetings, where theatre space and ICU provision were allocated across specialties; and the occasional reprioritization of patients within a specialty, in light of the theatre space secured at the cross-specialty meetings.

There was variation in surgeons’ reports of how they managed the first stage of within-specialty prioritization. Although most reported adhering to NHS England’s suggested priority grouping for patients with colorectal cancer (can be delayed for 10–12 weeks), some reported deviating from it (*Fig. S3*, E2).

A dimension of the decision-making reported by several interviewees concerned the doctor–patient relationship and how this could shape priority status judgement. Some referred to the temptation to inflate a patient’s priority based on emotional attachment, which complicated their objective assessment (*Fig. S3*, E3). Interviewees not only reported making decisions about whose operation could safely be delayed, but also referred to decision-making around which patients would be safer if surgery were delayed. Many noted that they had been keen to defer surgery for patients in whom the risks of operating during the pandemic were thought to outweigh the benefits (*Fig. S3*, E4), and that these decisions had been made jointly with the patient (*Fig. S3*, E5).

Despite some challenges, stage 1 of the prioritization process was generally relayed as a collaborative activity within the multidisciplinary team (*Fig. S3*, E6). By contrast, stage 2—cross-specialty prioritization—was relayed as a more challenging process. With theatre and ICU facilities limited, prioritization needed to occur among patients from different specialties who had often been assigned the same priority status (*Fig. S3*, E7). Informants with experience of these cross-specialty deliberations recalled numerous factors informing prioritization at this level, including efficiency considerations (for example patient throughput, linked to an operation’s complexity and time requirements) (*Fig. S3*, E8), prognosis (*Fig. S3*, E9), and postoperative resource needs (*Fig. S3*, E10). Difficulties appeared to stem from a lack of consensus around how to weight these factors.

The above issues were compounded by perceptions that decisions could be influenced by different groups’ conflicting agendas. For example, some referred to colleagues from other specialties as ‘gaming’, to prioritize their own patients (*Fig. S3*, E11). Cross-specialty decisions could also be affected by political issues relating to local management priorities. One informant for example, referred to their Trust prioritizing access to theatre lists for a particular specialty to address a performance issue and how this affected the colorectal team (*Fig. S3*, E12).

The complexity of the individual and cross-specialty prioritization processes were hard to disentangle from many accounts, as the processes were interlinked. Some informants described having to reprioritize patients within their own specialty, if there was misalignment with the theatre time secured at the cross-specialty meeting. For example, a surgeon reported having 12 patients who needed postoperative ICU facilities, but their secured list allowed access only to a single ICU bed per week. The colorectal team were thus required to rethink their prioritized list of patients, to include a combination of patients of higher and lesser priority to match the resource available.

Overall, surgical teams’ experiences of prioritizing patients were framed as an iterative, often unclear process, imbued with challenges and discomfort. Although this was key to managing demand, informants also discussed another type of decision-making, involving assessments about whether non-surgical treatment might be more appropriate for a patient. As discussed below, these decisions required adaptations to established practice, driven by the new risk/benefit profile of interventions.

#### Adapting patient treatment plans

Surgeons reported making adaptations to the operative procedure—a strategy that was often discussed in the context of national guidance, which recommended minimizing the burden on ICU services^7^. Several informants reported a shift towards performing more temporary end stomas, to reduce the duration of the procedure and increase patient throughput during limited theatre time (*Fig. S4*, E1). Strategic use of resources was also apparent in some informants’ explanations of how a shift away from performing anastomoses may reduce complications and need for ICU care (*Fig. S4*, E2). Increased use of stomas was not, however, routinely pursued by all (*Fig. S4*, E3).

Another change to usual practice reported by several interviewees was greater use of non-surgical treatments—specifically, neoadjuvant radiotherapy to delay surgery, as recommended in guidelines^6^ (*Fig. S4*, E4). Reflecting on perceived positive outcomes, surgeons considered that this adaptation might have implications for future routine practice (*Fig. S4*, E5).

#### Changing surgical team working practices

Altering working practices to optimize limited surgical capacity was reported. This included implementing the recommended dual-consultant operating^6^, and employing a team-based approach to managing operating lists.

Dual-consultant operating was discussed as a strategy to reduce theatre time, and a necessity, where surgical trainees had been redeployed. Most practitioners’ experience of the move to dual-consultant operating was positive and framed as unavoidable, but some expressed concern about increasing staff exposure to coronavirus (*Fig. S5*, E1).

The shift to a team-based approach to managing lists was more controversial. This was a process whereby a patient would be operated on by the next available skilled surgeon, not necessarily the surgeon who conducted the preoperative consultations. Some expressed concerns that this disrupted the doctor–patient relationship (*Fig. S5*, E2) and/or voiced discomfort around operating on patients they had only met on the day of surgery (*Fig. S5*, E3). As with the prioritization strategies, the team-based approach represented a change to practice that was emotive to some, requiring surgeons to adapt to new ways of working to manage the imbalance between resource and demand.

## Discussion

Using colorectal cancer surgery as a case study, this work investigated how surgical teams managed to uphold surgical services in the acute phases of the COVID-19 pandemic. Surgical teams’ actions coalesced around four overarching strategies: relocating surgical services; prioritizing patients within and across surgical specialties; adapting patient treatment plans; and introducing changes to surgical team working practices. Of these, prioritization and changes to team working were the most challenging to implement.

This study has several strengths and limitations. A key strength is the use of qualitative methods to generate rich understanding of practitioners’ experiences, motivations, and behaviours, insights that would have been challenging to capture quantitatively. Although the study was UK-based, rich descriptions help readers make assessments about the transferability to other healthcare systems. Recollection bias is always a risk with interviews, although the themes derived from multiple accounts lend confidence to the credibility of findings. The sample would, however, have benefited from inclusion of a wider array of healthcare professionals.

A key finding was the complexity and emotional effort of making prioritization decisions in practice. This fits with the work of Ives and Huxtable^12^, who anticipated the psychological and moral discomfort frontline practitioners were likely to experience. Emanuel *et al.*^13^ proposed lessening this burden by developing prioritization guidelines to be applied by an independent stakeholder group, including ethicists. An attempt to manage prioritization decisions in this way is reported by Catton *et al.*^14^. They concluded that a group, established in response to COVID-19, facilitated prioritization of patients based on clinical and ethical grounds, enabling surgery to continue during the pandemic.

Use of independent-sector provision was not challenge-free, but early concerns about staff skills and availability of ICU facilities were surmountable. Modifications to usual treatment plans enabled surgery to be delayed, easing pressure on operating facilities. The reports of increased neoadjuvant therapy use concur with previous work^8^. Colorectal teams were not alone in pursuing this strategy. Dave *et al.*^15^, for example, reported on altered management of patients with breast cancer. With an apparent increased use of non-surgical treatment, evidence on long-term patient outcomes is warranted, and could have implications for future routine care.

A shift to team surgery, although accepted on pragmatic grounds, proved emotive. Some surgeons voiced unease with performing surgery in a riskier than usual context, not having had time to try to develop a trusting relationship with the patient. The study findings align with the considerable body of work focusing on the significance of the surgeon–patient relationship as a necessary foundation for the responsibility that a surgeon assumes when operating^16^. The patient perspective on treatment during the pandemic is the subject of a forthcoming CONSIDER-19 paper.

The suite of early national guidance received mixed reception from study participants. Critique centred on timing, volume, robustness of the evidence base, and lack of alignment. In their quantitative evaluation of surgeons’ practice against UK national guidance, Byrne *et al.*^10^ found patchy levels of adherence. The findings of the present study help explain the patterns identified. Reliance on expert opinion was perhaps unavoidable in this early phase of the pandemic and was not unique to UK guidance. The COVIDSurg Collaborative^17^ conducted an international review of surgical guidance for surgeons and concluded that most recommendations were based on expert opinion. Although evidence generation takes time, concerns around volume and alignment can be addressed with a greater focus on coordination among lead organizations, across and within surgical subspecialties.

The findings have implications for both future medical emergencies and the recovery stages of the pandemic. First, better structural and ethical support is needed for surgeons tasked with prioritizing patients. Second, fuller consideration could be given to identifying and addressing the barriers and facilitators to drawing on independent-sector provision should access to NHS surgical capacity be restricted. Third, bodies responsible for producing guidance should commit to streamlining the quantity of guidance produced.

Prioritization decisions have and will always be needed in healthcare^18^. COVID-19 pressures have arguably exposed more frontline healthcare professionals to these contentious decisions. This study not only has implications for responses to acute resource constraint, but highlights opportunities to engage a wider array of stakeholders in priority-setting discussions in healthcare. For more detailed and comprehensive insights, future research could compare approaches to prioritization across different specialties, and whether and how these evolved as the COVID-19 pandemic progressed. The learning is likely to be beneficial as healthcare systems with limited physical and staffing resources tackle lengthy waiting lists during the COVID-19 recovery phase.

## Supplementary Material

znac371_Supplementary_Data

## Data Availability

The data set (transcripts) informing this study are available on request, by contacting carmel.conefrey@bristol.ac.uk. The data have not been uploaded to a public repository owing to privacy concerns, but the authors will be able to consider specific requests on an individual basis.
