# Erratum to: Managing surgical demand when needs outstrip resource: qualitative investigation of colorectal cancer surgery provision in the first wave of the COVID-19 pandemic

**DOI:** 10.1093/bjs/znac459

**Published:** 2023-01-04

This is an erratum to: Carmel Conefrey, Cynthia Ochieng, Christin Hoffmann, Daisy Elliott, Kerry Avery, Joanne Bennett, Natalie Blencowe, Sarah Duff, James Kinross, Angus McNair, David Messenger, Anne Pullyblank, Baljit Singh, Anni King, Sarah E Squire, Jane Blazeby, Barry Main, Leila Rooshenas, Managing surgical demand when needs outstrip resource: qualitative investigation of colorectal cancer surgery provision in the first wave of the COVID-19 pandemic, *British Journal of Surgery*, Volume 110, Issue 1, January 2023, Pages 92–97, https://doi.org/10.1093/bjs/znac371

In the originally published version, the third and twelfth authors’ names were mis-spelled.

These have been corrected online.

