# Supplementary material for: Managing surgical demand when needs outstrip resource: qualitative investigation of colorectal cancer surgery provision in the first wave of the COVID-19 pandemic
Source: Br J Surg. 2022 Nov 7;110(1):92–7. doi: 10.1093/bjs/znac371 (PMC10364543; doi:10.1093/bjs/znac371)
Supplement: znac371_Supplementary_Data [file znac371_supplementary_data.docx]

**Supplementary Material**

**Managing surgical demand when needs outstrip resource: qualitative investigation of colorectal cancer surgery provision in the first wave of the COVID-19 pandemic.**

- Dr Carmel Conefrey, Senior Research Associate, Population Health Sciences, University of Bristol – corresponding author.
- Dr Cynthia Ochieng, Senior Research Associate, Population Health Sciences, University of Bristol
- Dr Christin Hoffman, Senior Research Associate, Population Health Sciences, University of Bristol
- Dr Daisy Elliott, Research Fellow, Bristol Medical School, Population Health Sciences, University of Bristol
- Dr Kerry Avery, Senior Lecturer in Health Services Research, Centre for Surgical Research, Bristol Medical School, Population Health Sciences, University of Bristol
- Ms Joanne Bennett, Consultant Colorectal Surgeon, Gloucestershire Royal Hospitals NHS Foundation Trust
- Dr Natalie Blencowe, MRC Clinician Scientist and Linder Foundation Associate Professor in Clinical Trials and Honorary Consultant Upper GI Surgeon, University of Bristol & University Hospitals Bristol and Weston Foundation Trust
- Mrs Sarah Duff, Consultant General and Colorectal Surgeon, Manchester University NHS Foundation Trust
- Mr James Kinross, Consultant Colorectal Surgeon and Senior Lecturer in Colorectal Surgery, Imperial College Healthcare NHS Trust
- Mr Angus McNair, NIHR Clinician Scientist, Population Health Sciences, University of Bristol, North Bristol NHS Trust
- Mr David Messenger, Consultant Colorectal Surgeon, University Hospitals Bristol and Weston NHS Foundation Trust
- Ms Anne Pullybank, Consultant Colorectal Surgeon, North Bristol NHS Trust
- Mr Baljit Singh, Consultant Colorectal Surgeon and Honorary Associate Professor, University Hospitals Leicester
- Ms Anni King, Senior Research Associate, Population Health Sciences, University of Bristol
- Mrs Sarah Squire, Patient Representative and member of the Association of Coloproctology of Great Britain and Ireland Patient Liaison Group, and PPI member on the NHS Specialised Colorectal Clinical Reference Group
- Professor Jane Blazeby, Professor of Surgery and Honorary Consultant Surgeon, University of Bristol & University Hospitals Bristol and Weston Foundation Trust
- Mr Barry Main, Consultant Senior Lecturer in Oral and Maxillofacial Surgery, Bristol Dental School, University of Bristol
- Dr Leila Rooshenas, Senior Lecturer in Qualitative Health Sciences, Population Health Sciences, University of Bristol

**Corresponding Author:** Dr Carmel Conefrey,

[carmel.conefrey@bristol.ac.uk](mailto:carmel.conefrey@bristol.ac.uk),

Dr. Carmel Conefrey

Senior Research Associate for Quintet Programme

Population Health Sciences

Bristol Medical School 
University of Bristol

Canynge Hall

39 Whatley Rd

Bristol

BS8 2PS

**Appendix S1**

**Part 1 (Context) Interview Extracts - Capacity for surgery**

E1: *No new cancers at all that week, which is unprecedented. That's unheard of. That's not because they're not there; it's just simply because we're not diagnosing it. (Surgeon 06 )*

E2 *People weren’t actually going to their GP, so the numbers of patients dropped right from the very beginning of that pathway.* *(Colorectal nurse specialist 03)*

E3 *We're not seeing a lot of early cancers because it's typically the screening cancers that diagnose cancers in a very early stage. (Surgeon 06)*

E 4 *Everything stopped. [Hospital X] had a really high COVID rate, so everything stopped. There was no endoscopy. There was no CT scanning. There were no CT pneumocolons… for about a month. Yes, everything ceased. (Surgeon 25)*

E5 *Many patients didn't want to come to hospital for investigations. Many were frightened to come or anxious and simply just didn't turn up. (Surgeon 06)*

*E 6 It [theatre provision] was crippled very quickly surgically because they took over the theatres for ventilation. (Surgeon 10)*

*E7 We had limitations imposed on us because of the overall response, i.e. the expansion of ICU into recovery, which gazumped our future throughput and the reallocation of nurses. (Surgeon 16)*

*E8 My anaesthetists had gone, they were busy. They were looking after ventilated patients. (Surgeon 07)*

*E9 We also had a situation where some colleagues, because of other underlying medical conditions, were shielding. So we actually had reduced personnel. (Surgeon 03)*

**Figure S1: Part 1 Interview extracts - National Guidance**

*E1 We took onboard very seriously… I think correctly at the time. In retrospect maybe we didn’t need to. But at the time we followed the College of Surgeons guidelines and the PHE (Public Health England) guidelines*. *(Surgeon 23)*

*E2 I think the professional bodies kept them specific enough, but vague enough to appreciate that everyone's process locally was slightly different… I think they were very appreciative that people were just in different circumstances. (Surgeon 17)*

*E3 That, for me, is a slight frustration, that we still end up with local implementation of national guidance, which I think is an issue, really, as a surgeon. Because especially in the beginning, when people were frightened and really didn't know how this was going to pan out, knowing that you were wearing a mask and a plastic apron, versus the people down the road, who were practically in hazmat suits, that adds an additional layer of stress. (Surgeon 01)*

*E4 My personal view was that there was very little evidence base. It was more personal experience and experience of individuals and a society view. (Surgeon 03)*

*E5 It was coming from everywhere, and the Royal College of Surgeons, unhelpfully, released some overnight once, without any alignment to anybody else. That morning, we came in to find a registrar had refused to do a laparoscopic appendicectomy, because the Royal College of Surgeons have said laparoscopy could be bad. (Surgeon 01)*

*E6 You could literally look at the different bits of the guidelines and choose your favourite one. It was not helpful. (Surgeon 22)*

### **Figure S2: Part 2 Interview extracts - Relocation of surgical services**

*E1 Every single site had different rules on PPE….Where we came in and were used to a higher level of PPE in one site, it caused an awful lot of consternation and upset because we'd say, “We need a mask to do this,” and they’d say, “We're not allowed masks.”…..we had surgeons refusing, threatening to stop coming to work because they were being told they weren't allowed to wear masks. (Surgeon 10)*

*E2 The problem for us is that we kept sending patients there, and they’ve got such strict criteria that they…we were doing the surgery but their anaesthetists kept turning down all our patients. So for colorectal, it didn’t really work out too well, because of our patients. But I think for urology and breast, where they’re doing smaller procedures in fitter patients, they got more things done. (Surgeon 01)*

*E3 There were all the issues with the amount of cover we had up at this (Independent sector) hospital, because there was resident cover, as you would expect, but it wasn’t quite of the level we would have in (own NHS hospital). So, it wasn’t the sort of place where you could cope with someone becoming acutely unwell on a Sunday afternoon. (Surgeon 18)*

*E4 The private hospital made some quite fundamental changes to how they worked, in order to accommodate the amount of work we were sending them. So, for example, their ITU, which was basically a five-bedded, high dependency unit, then had to triple in size, which they managed to achieve. They had to upskill a lot of nurses on the wards. (Surgeon18)*

*E5 We don’t think that’s good for patient care sending them 70 miles to be operated on by a surgical team that they’ve never met. (Surgeon 24)*

### **Figure S3: Part 2 Interview extracts - Prioritisation within and across specialties**

*E1 It’s like the old phrase of ‘Playing God as a doctor’ is obviously outdated and very much so, particularly with the amount of evidence we use these days in everything we do but this really felt like finger in the air judgements and I felt uncomfortable…..It was new territory, I think. (Surgeon 19)*

*E2 There was the national guidance about, basically, all colorectal cases, including cancer, can wait more than three months. That was if you follow their algorithm. We immediately reclassified that and I wrote into one of the drafts: ‘Unless for cancer,’ so that we could keep doing cancer cases. (Surgeon 10)*

*E3 Sometimes, you know these patients. You have a relationship with them. You've already had a discussion with them and their families and you want the best for them, so the temptation is always to say it is more urgent than perhaps it is because you want them to go through. (Surgeon 06)*

*E4 So, a few very elderly, frail patients with relatively early cancers - who would have had surgery at the end of March/beginning of April - after discussion with the patient, we suggested that we delay for four to six weeks, so that they weren’t in hospital while the hospital was in a state of chaos. (Surgeon 04)*

*E5 A lot of patients were very much, “No, I don’t want to be coming in anyway.” … It was a discussion between the patients and the consultant, as to whether they went ahead. (Colorectal nurse specialist 01)*

*E6 We were given a degree of capacity and we were asked to prioritise our patients. Our MDT lead went through and said, "Look, this is the priority that I think..." Then, he asked the rest of the MDT to comment. Collectively, between us, we ranked patients in order of urgency. Then, we said, "Look, we think these are the absolute priority. (Surgeon 06).*

*E7 There was a meeting every week where there were potentially four theatres per day, shared between multiple specialties and representatives from each specialty would go to a meeting with the cancer lead and we’d try and work out which… who should get the theatres. (Surgeon 19)*

*E8 We were thinking more along the lines of maximum benefit for minimum resource. So, rather than doing a complex big case all day when we could do three more simple curative cases, we had to aim for that. (Surgeon 19)*

*E9 It was when we were up against, for instance, a patient (who) needed a pancreatic resection for instance where the prognosis we know of pancreatic cancer is terrible, even when you operate and they’re a bit more resource heavy. That’s when you get to difficult discussions about who should get the resource (Surgeon 19)*

*E10 If you do an oesophagus, it takes 2 weeks before they go home or 10 days at best. If you do a right hemi [hemicolectomy], it's 3 days (Surgeon 20)*

*E11 So, some surgeons did a bit of gaming, because we like to operate and they always think that their patients are the most important. (Surgeon 13)*

*E12 So, for a few weeks, there was a little bit of a tussle. Then, we had to really put our feet down and say, "Look, we need to get back to normalcy." (Surgeon 26)*

### **Figure S4: Part 2 Interview extracts – Adapting patient treatment plans**

*E1 In the first few weeks, there was a lot of consideration for doing end stomas rather than primarily joining the bowel together. Just so you could get the patient done quickly if you only had one theatre list and there were lots of other folk on it, (Surgeon 17)*

*E2 For about three months, the dictum was 'any colorectal resection will have a stoma'. We were not given a choice, really. We, as a team, decided that that's the only way we could continue with our operating...because if they had a leak and no ITU bed, then we were stuck. (Surgeon 26)*

*E3 Don’t take the tumour out and give them a bag, because that means a second operation. These are relatively low risk anastomoses. If it feels and looks good on the day, join the patient up. (Surgeon 7)*

*E4 We had a few patients who you could have gone either way with, we normally operate on them straightaway, but because it’s major surgery and it was the pandemic, instead we opted for radiotherapy for them….it’s radiotherapy and delay. (Surgeon 04)*

*E5 I think the consequence will be that short-course radiotherapy will become much more common and, perhaps, even the mainstay of care for rectal cancers, so that's the shift. (Surgeon 17)*

### **Figure S5: Part 2 Interview extracts – Changing surgical team working practices**

*E1 The idea of having two consultants expose themselves at the same time seemed crazy. (Surgeon 01)*

*E2 Some of the operations I do are long and difficult, and they have nasty complications. And I think you avoid complaints by communicating well with patients and them knowing you’ve got their interests at heart, and communicating with their families. It’s seeing them several times as well, it’s seeing them beforehand, it’s having the conversation about what the diagnosis is, it’s seeing them at the pre-op to take the consent, they’re familiar. Then when you see them on the day, it’s almost like they’re an old friend, I’ve seen them several times, we’ve already had that conversation. I’m not meeting them on the day and going over a whole load of new stuff when they’re all stressed. (Surgeon 04).*

*E3 So, it was definitely a bit more nerve wracking operating on a patient that you hadn’t actually met at the beginning. There was one patient I met on the day of surgery. There were two consultants there, so that was fine, but it was definitely uncomfortable. I was uncomfortable with it. (Surgeon 07)*

**Table S1. Overview of interview participants**

| **Region** | **Healthcare Professional** | **No. of Hospital Sites** |
| --- | --- | --- |
| South West England | 4 Surgeons  3 Colorectal Nurse Specialists  1 Stoma Nurse | 3 |
| London | 2 Surgeons | 1 |
| East of England | 1 Surgeon | 1 |
| East Midlands | 3 Surgeons | 3 |
| North West England | 7 Surgeons  1 Gastroenterologist | 4 |
| South Wales | 1 Surgeon | 1 |
| Lothian, Scotland | 1 Surgeon | 1 |
| Greater Glasgow, Scotland | 2 Surgeons | 2 |
| National Organisation | 1 Surgeon | NA |
